# Repurposing existing medications for coronavirus disease 2019: protocol for a rapid and living systematic review

**DOI:** 10.1186/s13643-021-01693-7

**Published:** 2021-05-07

**Authors:** Benjamin P. Geisler, Lara Zahabi, Adam Edward Lang, Naomi Eastwood, Elaine Tennant, Ljiljana Lukic, Elad Sharon, Hai-Hua Chuang, Chang-Berm Kang, Knakita Clayton-Johnson, Ahmed Aljaberi, Haining Yu, Chinh Bui, Tuan Le Mau, Wen-Cheng Li, Debbie Teodorescu, Ludwig Christian Hinske, Dennis L. Sun, Farrin A. Manian, Adam G. Dunn

**Affiliations:** 1 Ludwig Maximilian University, Marchionistr. 15, 81377 Munich, Germany; 2 Massachusetts General Hospital/Harvard Medical School, Boston, MA USA; 3 McGill University, Montreal, Canada; 4 McDonald Army Health Center, Fort Eustis, VA USA; 5 Wirral University Teaching Hospital NHS Foundation Trust, Wirral, UK; 6 Royal North Shore Hospital, Sydney, NSW Australia; 7 University of New South Wales, Sydney, NSW Australia; 8 University Hospital for Infectious Disease Zagreb “Dr. Fran Mihaljevic”, Zagreb, Croatia; 9 National Cancer Institute, Bethesda, MD USA; 10 Chang Gung Memorial Hospital/Chang Gung University, Taoyuan, Taiwan; 11 National Taipei University of Technology, Taipei, Taiwan; 12 Geisinger Medical Center, Danville, PA USA; 13 Proof Biotechnologies, Inc., Columbia, SC USA; 14 Portmore Hospital Complex, Portmore, Jamaica; 15 Brigham and Women’s Hospital, Boston, MA USA; 16 University of California San Francisco Medical Center, San Francisco, CA USA; 17 Massachusetts Institute for Technology, Cambridge, MA USA; 18 Institute of High Performance Computing, Singapore, Singapore; 19 California Polytechnic State University, San Luis Obispo, CA USA; 20 University of Sydney, Sydney, NSW Australia

**Keywords:** COVID-19, SARS-CoV2, Severe acute respiratory syndrome, SARS, Middle East respiratory syndrome Coronavirus, MERS-CoV, Repurposed, Repurposing

## Abstract

**Background:**

Coronavirus disease 2019 (COVID-19) has no confirmed specific treatments. However, there might be in vitro and early clinical data as well as evidence from severe acute respiratory syndrome and Middle Eastern respiratory syndrome that could inform clinicians and researchers. This systematic review aims to create priorities for future research of drugs repurposed for COVID-19.

**Methods:**

This systematic review will include in vitro, animal, and clinical studies evaluating the efficacy of a list of 34 specific compounds and 4 groups of drugs identified in a previous scoping review. Studies will be identified both from traditional literature databases and pre-print servers. Outcomes assessed will include time to clinical improvement, time to viral clearance, mortality, length of hospital stay, and proportions transferred to the intensive care unit and intubated, respectively. We will use the GRADE methodology to assess the quality of the evidence.

**Discussion:**

The challenge posed by COVID-19 requires not just a rapid review of drugs that can be repurposed but also a sustained effort to integrate new evidence into a living systematic review.

**Trial registration:**

PROSPERO 2020 CRD42020175648

**Supplementary Information:**

The online version contains supplementary material available at 10.1186/s13643-021-01693-7.

## Background

The coronavirus disease 2019 (COVID-19) pandemic represents one of the deadliest and economically most consequential outbreaks in around 100 years [1, 2]. To date, only remdesivir (Gilead Sciences) has shown to possibly lower the time to recovery [3, 4]. In the past, developing antiviral agents has taken 5.9 years on average [5]. In the current crisis, it appears feasible that this timeline will be significantly reduced if there were early signs of efficacy. Moreover, a candidate drug could be offered emergency use authorization by the U.S. Food and Drug Administration and similar designations by regulatory authorities across the world, as was the case with remdesivir.

Drug repurposing has been a successful strategy for a variety of therapeutic areas [6]. In the case of COVID-19, compounds of interest include those that have previously been found to either be clinically efficacious or have in vitro activity against the coronaviruses that cause severe acute respiratory syndrome (SARS) and Middle Eastern respiratory syndrome (MERS), which share significant structural similarities with SARS-CoV-2, the virus that causes COVID-19 [7, 8]. However, the existing clinical and preclinical research studying these viruses as a whole has never been systematically reviewed. Information on drugs potentially active against COVID-19 is expected to change rapidly as the results of ongoing and future studies become available. It is crucial that both researchers and health care providers are able to access the optimum and most up-to-date information to inform future or ongoing studies and provide clinical care.

The objectives for the current protocol are (1) to systematically review which existing medications have shown to be potentially effective against SARS or MERS and could therefore be potentially repurposed; (2) to present the early evidence that supports testing readily available drugs against SARS-CoV-2; (3) to provide the best available level of evidence for the efficacy of each individual candidate drug; and (4) to report harmful effects associated with use of these drugs.

## Methods/design

This protocol specifies the conduct and reporting of a systematic review in compliance with the Preferred Reporting Items for Systematic Review and Meta-Analysis Protocols 2015 statement (PRISMA-P) [9]. The protocol has been registered with the International Prospective Register of Systematic Reviews (PROSPERO) and assigned the identifier CRD42020175648. This is intended as a living systematic review that will be continuously updated via semi-automated approaches, and updates will be published intermittently based on triggers that include substantial changes to the evidence base for any included intervention.

### Data sources

Bibliographical databases for literature search include Medline (via PubMed), Embase, the International Clinical Trials Registry Platform (ICTRP), ClinicalTrials.gov (for nonrandomised studies and trials with summary results), as well as the following preprint servers: MedRxiv, BioRxiv, chemRxiv, Preprints.org, and the Chinese-language server ChinaXiv. Our search strategy combines terms for COVID-19, SARS, MERS, and their causative agents with drug names, based on the following eligibility criteria (see Additional file 2: Appendix).

### Eligibility criteria

All clinical, in vitro, and animal studies that test interventions used to treat diseases associated with SARS-CoV-2, SARS-CoV, or MERS-CoV are eligible for inclusion in the systematic review.

### Study design

Single-arm and controlled studies with or without randomisation as well as open-label or blinded designs will be included. Future clinical studies should be focused on agents that were found to be promising in in vitro or in animal studies, and these should be included here as we are aiming to identify potentially effective treatments.

### Interventions

The interventions we studied were derived from a scoping review by the World Health Organisation (WHO) [10]. They encompass both single-agent and combination regimens with one or more of the following agents: atazanavir, azithromycin, baloxavir marboxil, baricitinib, bevacizumab, chloroquine, colchicine, darunavir/cobicistat, emtricitabine/tenofovir, enisamium iodide, favipiravir (T-705), fingolimod, ganciclovir, hydroxychloroquine, indinavir, lopinavir/ritonavir, mycophenolic acid/mofetil, nelfinavir, niclosamide, nitazoxanide, nitric oxide, novaferon, oseltamivir, pirfenidone, quercetin, remdesivir (GS-5734), ribavirin, ruxolitinib, sirolimus, sofosbuvir, tocilizumab, thymosin alpha-1, triazavirin, and umifenovir. We have additionally included glucocorticosteroids (with or without mineralocorticoids), interferons, statins, and convalescent serum or plasma.

### Comparators, participants, follow-up periods, and languages

For clinical studies, eligible studies include patients with mild-to-moderate or severe disease in both outpatient and inpatient settings, with the latter including intensive care unit (ICU) and non-ICU patients. For clinical studies to be included, participants must not just have symptoms compatible with COVID-19 but either laboratory confirmation of infection of respective viruses—severe acute respiratory syndrome coronavirus 2 (SARS-CoV-2), severe acute respiratory syndrome coronavirus (SARS-CoV), or Middle Eastern Respiratory Syndrome Coronavirus (MERS-CoV)—or be presumed to be infected on clinical grounds after careful deliberation. No restrictions on minimum follow-up periods or language of the articles are included. Our team includes native speakers of various languages, and additional translator services will be used as needed.

Animal and in vitro studies are eligible for inclusion if they seek to provide evidence on the potential human use of the interventions for the conditions described above. These studies will be summarised separately from the clinical studies in a summary of findings table and visualised together with clinical studies illustrate the current pipeline of evidence for early investigations into new treatments.

### Outcomes

Studies are eligible if they include clinical endpoints related to time to clinical improvement, time to viral clearance, mortality, hospital admission or transfer to a higher level of care, including transfer to an ICU or ICU-like setting and total and ICU length of stay, proportion of intubations, length of mechanical ventilation, normalization of selected laboratory data (such as C-reactive protein, d-dimer, lactate dehydrogenase, ferritin, and lymphocyte count), and adverse events.

### Study selection, data management, and data extraction

Two reviewers will use the same eligibility criteria to evaluate the studies. Conflicts will be resolved by discussion. Data management for the study selection process will occur in Covidence (Veritas Health Innovation, Melbourne, Australia). Study data will be extracted by one reviewer and verified by a second reviewer. In addition to the outcome measures, the following characteristics of the verified RCTs will be extracted: (1) reference details (including the first author’s last name and the publication year); (2) country of origin; (3) the specific coronavirus studied (SARS-CoV-2, SARS-CoV, or MERS-CoV); (4) type of cell or animal studied (applicable only to in vitro or animal study); (5) blinded versus open-label design (applicable to controlled studies); (6) inclusion and exclusion criteria; (7) baseline patient characteristics, in particular duration since onset of symptoms, outpatient versus non-ICU versus ICU setting, and mild-to-moderate versus severe disease; (8) intervention(s) studied; (9) control(s, if any); (10) total number of participants; (11) primary outcome; (12) secondary outcome(s); (13) results; and (14) conclusions. Corresponding authors will be contacted for clarification where necessary.

### Quality and risk of bias assessment

The quality of the available evidence will be evaluated using the Grading of Recommendations Assessment, Development and Evaluation (GRADE) framework [11] and applied separately for each intervention with one or more included studies in humans. Risk of bias will be assessed for any randomised trials using the revised Cochrane risk of bias tool [12], and the Risk of Bias in Nonrandomised Studies of Interventions (ROBINS-I) tool [11]. Animal and in vitro studies will not be assessed for risk of bias, and a narrative review of these studies will be done separately.

### Data synthesis and analysis

The extracted data will be collated and qualitatively synthesized via an interactive mechanism to select, sort, and filter columns for the results tables on our website (www.CovidDrugs.org). We will devise various pre-populated tables that can be viewed and downloaded as well. Tables that include a summary of findings will be developed for each treatment (incorporating multiple outcomes per table) and included on the website as well as in the Additional file 2: Appendix of the manuscript and its updates.

While the review is initially focused on mapping the status and quality of evidence for each of the treatments that are being tested for use in treating COVID-19, we will undertake safety and efficacy meta-analyses where possible. Where multiple controlled studies are available for a similar patient population, we will calculate a relative risk with a 95% confidence interval via a random-effects meta-analysis in RevMan, STATA, or the R metafor package. Heterogeneity will be assessed as τ^2^. Funnel plots and Egger’s test will be reported to assess publication bias.

### Living systematic review

COVID-19 is now endemic and there is a need to identify, review, critically appraise, and synthesize new studies in an ongoing way [13]. Following the first complete publication of the systematic review, it will be transformed into a living and open systematic review [14–17]. This includes automated search updates, semi-automated combining and de-duplicating all search results, and semi-automated updating of included study lists and summary tables made available on the website and in an extensible markup language (XML) stream. As new articles or registrations are identified by daily database searches, these are assigned to reviewers for screening using an alert system. Once screened, their relevance and inclusion status will be updated on the website and in the XML stream. The search terms will be reviewed intermittently and updated by consensus among the reviewer group during meetings, and at least quarterly.

The proposed living systematic review seeks to monitor the quality of evidence for repurposed drugs used in COVID-19. Our initial focus is on providing a platform for capturing relevant controlled studies as they are registered, completed, and reported, as well as animal and in vitro studies that may support the need for clinical studies. Synthesis is focused on visualising important information including the design, grading, risk of bias and extracted summary results. As results for more trials become available, the systematic review will be extended to include individual safety and efficacy meta-analyses for some of the interventions and outcomes. Where future updates include meta-analyses for individual treatments and outcomes, appropriate tests for heterogeneity and meta-biases will be applied.

In terms of dissemination, the completed review manuscript and intermittent updates (e.g., when the number of included studies changes, when a conclusion for any intervention changes, or for any other pre-defined triggers) will be posted to medRxiv. This ensures that each substantial update can be cited but the complete history and most recently available evidence is available in one location with a single digital object identifier (DOI).

We will adapt and use methods from natural language processing and crowd sourcing to support the review process, learning from recently developed tools and processes to add non-human and crowd-based screening and extraction [18–20]. We will open source our methods and results and intend to crowd source future review efforts, which may alter the number of authors on review updates.

## Discussion

This systematic review will summarize the emerging evidence on repurposed drugs for the treatment of COVID-19, extrapolating from early evidence for COVID-19 itself as well as SARS and MERS. The protocol extends beyond synthesizing evidence about potential treatment compounds that were identified in a scoping review by the WHO to incorporate pre-print servers and trial registries and to become an ongoing monitoring of the available evidence for each of the treatments. Initial syntheses are focused on mapping the evidence in terms of how much is available across animal, in vitro, and clinical studies, as well as the quality and risks of bias in any clinical studies. This protocol was written in accordance with the PRISMA-P statement (Additional file 1) and is registered with PROSPERO.

The challenge posed by COVID-19 requires not just a rapid review of drugs that can be repurposed but also a sustained effort to monitor the evidence base as it evolves. By incorporating animal and in vitro studies, the approach should be particularly useful in prioritizing candidates for testing in clinical studies and compare across the broad range of drugs that have been proposed. Finally, future investigators could benefit from preliminary estimates of efficacy for power and sample size calculations where appropriate, and their study designs could inform relevant clinical safety and efficacy endpoints as well as gaps in outcome measures that should be incorporated into future studies.

## Supplementary Information

**Additional file 1.** PRISMA-P 2015 Checklist.

**Additional file 2.** Appendix.

## Data Availability

All data and materials are available at www.CovidDrugs.org.

## Abbreviations

COVID-19: Coronavirus disease 2019
GRADE: Grading of Recommendations Assessment, Development and Evaluation
MERS: Middle Eastern respiratory syndrome
MERS-CoV: Middle Eastern respiratory syndrome coronavirus
PRISMA-P: Preferred Reporting Items for Systematic Review and Meta-Analysis Protocols
PROSPERO: International Prospective Register of Systematic Reviews
SARS: Severe acute respiratory syndrome
SARS-CoV: Severe acute respiratory syndrome coronavirus
SARS-CoV-2: Severe acute respiratory syndrome coronavirus 2
XML: Extensible Markup Language
WHO: World Health Organisation
