# Supplementary material for: Repurposing existing medications for coronavirus disease 2019: protocol for a rapid and living systematic review
Source: Syst Rev. 2021 May 7;10:143. doi: 10.1186/s13643-021-01693-7 (PMC8103882; doi:10.1186/s13643-021-01693-7)
Supplement: Supplementary file 2 — Additional file 2. Appendix. [file 13643_2021_1693_MOESM2_ESM.docx]

# Appendix

## PubMed

("COVID-19" [Supplementary Concept] or
"covid-19*"[tiab] or
"covid19*"[tiab] or
"sars-cov2*"[tiab] OR
"2019-nCoV"[tiab] OR
((novel*[tiab] OR 2019[tiab]) AND ("Coronavirus Infections"[Mesh] OR Coronavirus*[tiab] OR "Corona-virus*"[tiab] OR (Corona*[tiab] and virus*[tiab]))) OR
"SARS Virus"[Mesh] OR
"Severe Acute Respiratory Syndrome*"[tiab] OR
"SARS-CoV"[tiab] OR
SARS*[tiab] OR
"Middle East Respiratory Syndrome Coronavirus"[Mesh] OR
"Middle East Respiratory Syndrome"[tiab] **OR**
"MERS-CoV*"[tiab] OR
MERS*[tiab])

AND

("arbidol" [Supplementary Concept] OR arbidol*[tiab] OR Umifenovir*[tiab] OR
"Azithromycin"[Mesh] OR Azithromycin*[tiab] OR
"baloxavir" [Supplementary Concept] OR baloxavir*[tiab] or
"baricitinib" [Supplementary Concept] OR baricitinib*[tiab] OR
"Bevacizumab"[Mesh] OR Bevacizumab[tiab] OR
"Chloroquine"[Mesh] OR Chloroquine*[tiab] OR
"Darunavir"[Mesh] OR Darunavir*[tiab] OR "Cobicistat"[Mesh] OR Cobicistat*[tiab] OR
"Emtricitabine, Tenofovir Disoproxil Fumarate Drug Combination"[Mesh] OR "Emtricitabine"[Mesh] OR Emtricitabine*[tiab] or Tenofovir*[tiab] OR
"enisamium" [Supplementary Concept] OR enisamium*[tiab] or
"favipiravir" [Supplementary Concept] OR favipiravir[tiab] OR
"Fingolimod Hydrochloride"[Mesh] OR Fingolimod*[tiab] OR
"Ganciclovir"[Mesh] OR Ganciclovir*[tiab] OR
"Hydroxychloroquine"[Mesh] OR Hydroxychloroquine*[tiab] OR " Hydroxy-chloroquine*[tiab]"
"Lopinavir"[Mesh] OR "lopinavir-ritonavir drug combination" [Supplementary Concept] OR Lopinavir*[tiab] OR
"Mycophenolic Acid"[Mesh] OR "Mycophenolic Acid"[tiab] OR "mycophenolate"[tiab] OR (Mycophenol*[tiab] AND Acid [tiab]) OR (Mycophenol*[tiab] AND Mofetil*[tiab]) OR (Mofetil[tiab] and Hydrochlorid*[tiab]) OR
"Nelfinavir"[Mesh] OR Nelfinavir*[tiab] OR
"Niclosamide"[Mesh] OR Niclosamide*[tiab] OR
"nitazoxanide" [Supplementary Concept] OR nitazoxanide*[tiab] OR
"Novaferon" [Supplementary Concept] OR Novaferon*[tiab] OR
"Oseltamivir"[Mesh] OR Oseltamivir*[tiab] OR
"pirfenidone" [Supplementary Concept] OR pirfenidone*[tiab] OR
"Quercetin"[Mesh] OR Quercetin*[tiab] OR
"remdesivir" [Supplementary Concept] OR remdesivir*[tiab] or
"Ribavirin"[Mesh] OR Ribavirin*[tiab] OR
"INCB018424" [Supplementary Concept] OR Ruxolitinib*[tiab] OR
"Sirolimus"[Mesh] OR Sirolimus*[tiab] OR
"Sofosbuvir"[Mesh] OR Sofosbuvir*[tiab] OR
"Thymalfasin"[Mesh] OR "thymosin alpha(1) (24-28)" [Supplementary Concept] OR Thymalfasin*[tiab] OR
"triazavirin" [Supplementary Concept] OR triazavirin*[tiab] OR
"tocilizumab" [Supplementary Concept] OR tocilizumab*[tiab] OR
"Steroids"[Mesh] or steroid*[tiab] or corticosteroid*[tiab] or "cortico-steroid*"[tiab] or "Hydrocortisone"[Mesh] or Hydrocort*[tiab] OR "Cortisone"[Mesh] OR Cortis*[Mesh] OR "Prednisone"[Mesh] OR "prednisone sodium succinate" [Supplementary Concept] OR Prednis*[tiab] OR "Prednisolone"[Mesh] "Methylprednisolone"[Mesh] OR "Methylprednisolone Acetate"[Mesh] OR "Methylprednisolone Hemisuccinate"[Mesh] OR Methylpred*[tiab] or "Methyl-pred*"[tiab] or "Dexamethasone"[Mesh] OR "Dexamethasone Isonicotinate"[Mesh] OR dexameth*[tiab] or "Fludrocortisone"[Mesh] OR "fludrocortisone acetate" [Supplementary Concept] OR fludrocort*[tiab] OR
"Interferon-alpha"[Mesh] OR "Interferon alpha-2"[Mesh] OR "Receptor, Interferon alpha-beta"[Mesh] or "interferon-alpha*"[tiab] or (interferon*[tiab] and alpha[tiab]) OR "interferon alfacon-1" [Supplementary Concept] OR (interferon*[tiab] AND ("alfacon-1"[tiab] OR alfacon*[tiab])) OR
"Interferon-beta"[Mesh] OR "Interferon beta-1b"[Mesh] OR "Interferon beta-1a"[Mesh] OR "interferon-beta*"[tiab] or (interferon*[tiab] and beta[tiab]) OR
"Interferon-gamma"[Mesh] OR "interferon-gamma*"[tiab] or (interferon*[tiab] and gamma[tiab]) OR
"Atorvastatin Calcium"[Mesh] OR "atorvastatin*"[tw] OR "Rosuvastatin Calcium"[Mesh] OR "rosuvastatin*"[tw] OR "Simvastatin"[Mesh] OR "simvastatin acid" [Supplementary Concept] OR "simvastatin*"[tw] OR "Pravastatin"[Mesh] OR "Pravastatin*"[tw] OR "fluvastatin"[Supplementary Concept] OR "fluvastatin*"[tw] OR "fluindostatin*"[tw] OR
"Lovastatin"[Mesh] OR "Lovastatin*"[tw] OR "mevinolin*"[tw] OR "pitavastatin"[Supplementary Concept] OR "pitavastatin*"[tw] OR "statin*")

## Embase

| No. | Query | Results |
| --- | --- | --- |
| #53 | #47 AND #52 | **1975** |
| #52 | #48 OR #50 OR #51 | **13623** |
| #51 | 'middle east respiratory syndrome' OR 'middle east respiratory syndrome coronavirus' | **2675** |
| #50 | 'severe acute respiratory syndrome' OR 'sars-related coronavirus' OR 'sars coronavirus' | **11074** |
| #49 | #47 AND #48 | **101** |
| #48 | #1 OR #2 OR #3 OR #4 OR #5 OR #6 OR #11 | **1006** |
| #47 | #12 OR #13 OR #14 OR #15 OR #16 OR #17 OR #18 OR #19 OR #20 OR #21 OR #22 OR #23 OR #24 OR #25 OR #26 OR #27 OR #28 OR #29 OR #30 OR #31 OR #32 OR #33 OR #34 OR #35 OR #36 OR #37 OR #38 OR #39 OR #40 OR #41 OR #42 OR #43 OR #44 OR #45 OR #46 | **1841871** |
| #46 | 'mineralocorticoid' OR 'fludrocortisone' OR 'fludrocortisone acetate' OR 'fludrocortisone related compounds' OR 'fludrocortisone therapeutic use' | **24803** |
| #45 | 'steroid' OR 'corticosteroid' OR 'prednisone' OR 'prednisone acetate' OR 'prednisone therapeutic use' OR 'prednisone therapy' OR 'prednisolone' OR 'prednisolone derivative' OR 'prednisolone acetate' OR 'methylprednisolone' OR 'methylprednisolone sodium succinate' OR 'methylprednisolone acetate' OR 'methylprednisolone aceponate' OR 'dexamethasone' OR 'dexamethasone derivative' | **1012020** |
| #44 | 'hydroxymethylglutaryl coenzyme a reductase inhibitor' OR 'atorvastatin' OR 'rosuvastatin' OR 'simvastatin' OR 'pravastatin' OR 'fluindostatin' OR 'mevinolin' OR 'pitavastatin' | **144403** |
| #43 | 'interferon' OR 'alpha interferon' OR 'alpha2b interferon' OR 'beta interferon' OR 'beta1a interferon' OR 'interferon beta serine' OR 'gamma interferon' | **383685** |
| #42 | 'riamilovir' | **18** |
| #41 | 'thymosin alpha1' | **1115** |
| #40 | 'tocilizumab' | **11636** |
| #39 | 'sofosbuvir' | **9123** |
| #38 | 'rapamycin' | **99624** |
| #37 | 'ruxolitinib' | **4597** |
| #36 | 'ribavirin' OR 'peginterferon alpha2a plus ribavirin' OR 'peginterferon alpha2b plus ribavirin' OR 'rebetron' OR 'ribavirin derivative' OR 'taribavirin' | **40884** |
| #35 | 'remdesivir' | **82** |
| #34 | 'quercetin' | **36679** |
| #33 | 'pirfenidone' | **3442** |
| #32 | 'oseltamivir' | **11044** |
| #31 | 'novaferon' | **13** |
| #30 | 'nitazoxanide' | **1936** |
| #29 | 'niclosamide' | **2161** |
| #28 | 'nelfinavir' | **9928** |
| #27 | 'mycophenolate mofetil' OR 'mycophenolic acid' | **67223** |
| #26 | 'lopinavir plus ritonavir' OR 'lopinavir' | **12977** |
| #25 | 'hydroxychloroquine' OR 'hydroxychloroquine sulfate' | **24503** |
| #24 | 'ganciclovir' | **25145** |
| #23 | 'fingolimod' | **9447** |
| #22 | 'favipiravir' | **671** |
| #21 | 'enisamium iodide' | **4** |
| #20 | 'emtricitabine plus tenofovir disoproxil' OR 'emtricitabine' | **13412** |
| #19 | 'darunavir' OR 'cobicistat plus darunavir' | **6217** |
| #18 | 'chloroquine' | **42835** |
| #17 | 'bevacizumab' | **58194** |
| #16 | 'baricitinib' | **999** |
| #15 | 'baloxavir' OR 'baloxavir marboxil' | **164** |
| #14 | 'azithromycin' | **35638** |
| #13 | 'umifenovir' | **38** |
| #12 | 'arbidol' | **268** |
| #11 | #9 AND #10 | **318** |
| #10 | #7 OR #8 | **4257907** |
| #9 | 'coronaviridae' | **1272** |
| #8 | '2019' | **2889435** |
| #7 | 'novel' | **1589513** |
| #6 | '2019 novel coronavirus' | **289** |
| #5 | '2019-ncov' | **277** |
| #4 | 'sars-cov2' | **6** |
| #3 | 'covid-19' | **373** |
| #2 | 'covid19' | **1** |
| #1 | 'covid 19' | **373** |

Download RIS file

## Google Scholar

Keyword: Arbidol OR atazanavir OR Azithromycin OR Baloxavir OR Baricitinib OR Bevacizumab OR Chloroquine OR Colchicine OR Darunavir OR Emtricitabine OR Enisamium OR Favipiravir OR Fingolimod OR Ganciclovir OR Hydroxychloroquine OR Indinavir OR Lopinavir OR Mycophenolic OR Nelfinavir OR Niclosamide OR Nitazoxanide OR Novaferon OR Oseltamivir OR Pirfenidone OR Quercetin OR Remdesivir OR Ribavirin OR Ruxolitinib OR Sirolimus OR Sofosbuvir OR Tocilizumab OR Thymosin OR Triazavirin OR steroids OR steroid OR glucocorticosteroid OR glucocorticosteroids OR corticosteroid OR corticosteroids OR cortisone OR hydrocortisone OR prednisone OR prednisolone OR methylprednisolone OR dexamethasone OR fludrocortisone OR interferon OR atorvastatin OR rosuvastatin OR simvastatin OR pravastatin OR Fluvastatin OR fluindostatin OR lovastatin OR pitavstatin OR convalescent OR reconvalescent

AND title:

Covid-19

Covid19

Covid2019

Covid-2019

2019-ncov

sars-cov-2

SARS

Severe acute respiratory syndrome

MERS

Middle eastern respiratory syndrome

"novel coronavirus"

"2019 coronavirus"

"2019 coronavirus"

"coronavirus" "corona virus" "corona-virus" and limit years to 2019 and 2020

## ClinicalTrials.gov

**Condition or Disease:** covid-19 OR 2019-nCoV OR SARS-CoV-2 OR "2019 novel coronavirus" OR "severe acute respiratory syndrome coronavirus 2" OR mers OR "Middle East Respiratory Syndrome" " MERS-CoV" OR SARS OR "Severe acute respiratory syndrome"

Filter: “with results”

Darunavir Emtricitabine Enisamium Favipiravir Fingolimod Ganciclovir Hydroxychloroquine Indinavir Lopinavir Mycophenolic Nelfinavir Niclosamide Nitazoxanide Novaferon Oseltamivir Pirfenidone Quercetin Remdesivir Ribavirin Ruxolitinib Sirolimus Sofosbuvir Tocilizumab Thymosin Triazavirin steroids steroid glucocorticosteroid glucocorticosteroids corticosteroid corticosteroids cortisone hydrocortisone prednisone prednisolone methylprednisolone dexamethasone fludrocortisone interferon atorvastatin rosuvastatin simvastatin pravastatin Fluvastatin fluindostatin lovastatin pitavstatin convalescent reconvalescent serum plasma

## MedRxiv

for abstract or title "Arbidol atazanavir Azithromycin Baloxavir Baricitinib Bevacizumab Chloroquine Colchicine Darunavir Emtricitabine Enisamium Favipiravir Fingolimod Ganciclovir Hydroxychloroquine Indinavir Lopinavir Mycophenolic Nelfinavir Niclosamide Nitazoxanide Novaferon Oseltamivir Pirfenidone Quercetin Remdesivir Ribavirin Ruxolitinib Sirolimus Sofosbuvir Tocilizumab Thymosin Triazavirin steroids steroid glucocorticosteroid glucocorticosteroids corticosteroid corticosteroids cortisone hydrocortisone prednisone prednisolone methylprednisolone dexamethasone fludrocortisone interferon atorvastatin rosuvastatin simvastatin pravastatin Fluvastatin fluindostatin lovastatin pitavstatin convalescent reconvalescent" (match any words)

<https://www.biorxiv.org/search/%20abstract_title:Arbidol+atazanavir+Azithromycin+Baloxavir+Baricitinib+Bevacizumab+Chloroquine+Colchizine+Darunavir+Emtricitabine+Enisamium+Favipiravir+Fingolimod+Ganciclovir+Hydroxychloroquine+Indinavir+Lopinavir+Mycophenolic+Nelfinavir+Niclosamide+Nitazoxanide+Novaferon+Oseltamivir+Pirfenidone+Quercetin+Remdesivir+Ribavirin+Ruxolitinib+Sirolimus+Sofosbuvir+Tocilizumab+Thymosin+Triazavirin+steroids+steroid+glucocorticosteroid+glucocorticosteroids+corticosteroid+corticosteroids+cortisone+hydrocortisone+prednisone+prednisolone+methylprednisolone+dexamethasone+fludrocortisone+interferon+atorvastatin+rosuvastatin+simvastatin+pravastatin+Fluvastatin+fluindostatin+lovastatin+pitavstatin+convalescent+reconvalescent%20abstract_title_flags:match-any%20jcode:medrxiv%20numresults:75%20sort:relevance-rank%20format_result:standard>

Download the references and search for

- Covid-19
- Covid19
- Covid2019
- Covid-2019
- Covid
- novel coronavirus
- 2019 coronavirus
- Coronavirus
- corona
- 2019-ncov
- sars-cov-2
- SARS
- Severe acute respiratory syndrome
- MERS
- Middle eastern respiratory syndrome

## BioRxiv

for abstract or title "Arbidol atazanavir Azithromycin Baloxavir Baricitinib Bevacizumab Chloroquine Colchicine Darunavir Emtricitabine Enisamium Favipiravir Fingolimod Ganciclovir Hydroxychloroquine Indinavir Lopinavir Mycophenolic Nelfinavir Niclosamide Nitazoxanide Novaferon Oseltamivir Pirfenidone Quercetin Remdesivir Ribavirin Ruxolitinib Sirolimus Sofosbuvir Tocilizumab Thymosin Triazavirin steroids steroid glucocorticosteroid glucocorticosteroids corticosteroid corticosteroids cortisone hydrocortisone prednisone prednisolone methylprednisolone dexamethasone fludrocortisone interferon atorvastatin rosuvastatin simvastatin pravastatin Fluvastatin fluindostatin lovastatin pitavstatin convalescent reconvalescent" (match any words)

<https://biorxiv.org/search/%20abstract_title%3AArbidol%2Batazanavir%2BAzithromycin%2BBaloxavir%2BBaricitinib%2BBevacizumab%2BChloroquine%2BColchizine%2BDarunavir%2BEmtricitabine%2BEnisamium%2BFavipiravir%2BFingolimod%2BGanciclovir%2BHydroxychloroquine%2BIndinavir%2BLopinavir%2BMycophenolic%2BNelfinavir%2BNiclosamide%2BNitazoxanide%2BNovaferon%2BOseltamivir%2BPirfenidone%2BQuercetin%2BRemdesivir%2BRibavirin%2BRuxolitinib%2BSirolimus%2BSofosbuvir%2BTocilizumab%2BThymosin%2BTriazavirin%2Bsteroids%2Bsteroid%2Bglucocorticosteroid%2Bglucocorticosteroids%2Bcorticosteroid%2Bcorticosteroids%2Bcortisone%2Bhydrocortisone%2Bprednisone%2Bprednisolone%2Bmethylprednisolone%2Bdexamethasone%2Bfludrocortisone%2Binterferon%2Batorvastatin%2Brosuvastatin%2Bsimvastatin%2Bpravastatin%2BFluvastatin%2Bfluindostatin%2Blovastatin%2Bpitavstatin%2Bconvalescent%2Breconvalescent%20abstract_title_flags%3Amatch-any%20jcode%3Abiorxiv%20numresults%3A75%20sort%3Arelevance-rank%20format_result%3Astandard>

Download the references and search for

- Covid-19
- Covid19
- Covid2019
- Covid-2019
- Covid
- novel coronavirus
- 2019 coronavirus
- Coronavirus
- corona
- 2019-ncov
- sars-cov-2
- SARS
- Severe acute respiratory syndrome
- MERS
- Middle eastern respiratory syndrome

## ChemRxiv

Title contains:

- Covid-19 OR
- Covid19 OR
- 2019-ncov OR
- sars-cov-2 OR
- Severe acute Respiratory syndrome OR
- Middle Eastern Respiratory syndrome

AND Title contains:

- Arbidol OR atazanavir OR Azithromycin OR Baloxavir OR Baricitinib OR Bevacizumab OR Chloroquine OR Colchicine OR Darunavir OR Emtricitabine OR Enisamium OR Favipiravir OR Fingolimod OR Ganciclovir OR Hydroxychloroquine OR Indinavir OR Lopinavir
- Mycophenolic OR Nelfinavir OR Niclosamide OR Nitazoxanide OR Novaferon OR Oseltamivir OR Pirfenidone OR Quercetin OR Remdesivir OR Ribavirin OR Ruxolitinib OR Sirolimus OR Sofosbuvir OR Tocilizumab OR Thymosin OR Triazavirin OR steroids OR steroid
- glucocorticosteroid OR glucocorticosteroids OR corticosteroid OR corticosteroids OR cortisone OR hydrocortisone OR prednisone OR prednisolone OR methylprednisolone OR dexamethasone OR fludrocortisone OR interferon OR atorvastatin OR rosuvastatin
- simvastatin OR pravastatin OR Fluvastatin OR fluindostatin OR lovastatin OR pitavstatin OR convalescent OR reconvalescent

[https://chemrxiv.org/?q=%3Atitle%3A%20covid-19%20or%20%3Atitle%3A%20covid19%20or%20%3Atitle%3A%202019-ncov%20or%20%3Atitle%3A%20sars-cov2%20or%20%3Atitle%3A%20severe%20acute%20respiratory%20syndrome%20or%20%3Atitle%3A%20middle%20eastern%20respiratory%20syndrome%20and%20%3Atitle%3A%20Arbidol%20OR%20%3Atitle%3A%20atazanavir%20OR%20%3Atitle%3A%20Azithromycin%20OR%20%3Atitle%3A%20Baloxavir%20OR%20%3Atitle%3A%20Baricitinib%20OR%20%3Atitle%3A%20Bevacizumab%20OR%20%3Atitle%3A%20Chloroquine%20OR%20%3Atitle%3A%20Colchicine%20OR%20%3Atitle%3A%20Darunavir%20OR%20%3Atitle%3A%20Emtricitabine%20OR%20%3Atitle%3A%20Enisamium%20OR%20%3Atitle%3A%20Favipiravir%20OR%20%3Atitle%3A%20Fingolimod%20OR%20%3Atitle%3A%20Ganciclovir%20OR%20%3Atitle%3A%20Hydroxychloroquine%20OR%20%3Atitle%3A%20Indinavir%20OR%20%3Atitle%3A%20Lopinavir%20%3Atitle%3A%20Mycophenolic%20OR%20%3Atitle%3A%20Nelfinavir%20OR%20%3Atitle%3A%20Niclosamide%20OR%20%3Atitle%3A%20Nitazoxanide%20OR%20%3Atitle%3A%20Novaferon%20OR%20%3Atitle%3A%20Oseltamivir%20OR%20%3Atitle%3A%20Pirfenidone%20OR%20%3Atitle%3A%20Quercetin%20OR%20%3Atitle%3A%20Remdesivir%20OR%20%3Atitle%3A%20Ribavirin%20OR%20%3Atitle%3A%20Ruxolitinib%20OR%20%3Atitle%3A%20Sirolimus%20OR%20%3Atitle%3A%20Sofosbuvir%20OR%20%3Atitle%3A%20Tocilizumab%20OR%20%3Atitle%3A%20Thymosin%20OR%20%3Atitle%3A%20Triazavirin%20OR%20%3Atitle%3A%20steroids%20OR%20%3Atitle%3A%20steroid%20or%20%3Atitle%3A%20glucocorticosteroid%20OR%20%3Atitle%3A%20glucocorticosteroids%20OR%20%3Atitle%3A%20corticosteroid%20OR%20%3Atitle%3A%20corticosteroids%20OR%20%3Atitle%3A%20cortisone%20OR%20%3Atitle%3A%20hydrocortisone%20OR%20%3Atitle%3A%20prednisone%20OR%20%3Atitle%3A%20prednisolone%20OR%20%3Atitle%3A%20methylprednisolone%20OR%20%3Atitle%3A%20dexamethasone%20OR%20%3Atitle%3A%20fludrocortisone%20OR%20%3Atitle%3A%20interferon%20OR%20%3Atitle%3A%20atorvastatin%20OR%20%3Atitle%3A%20rosuvastatin%20or%20%3Atitle%3A%20simvastatin%20OR%20%3Atitle%3A%20pravastatin%20OR%20%3Atitle%3A%20Fluvastatin%20OR%20%3Atitle%3A%20fluindostatin%20OR%20%3Atitle%3A%20lovastatin%20OR%20%3Atitle%3A%20pitavstatin%20OR%20%3Atitle%3A%20convalescent%20OR%20%3Atitle%3A%20reconvalescent&searchMode=1](https://chemrxiv.org/?q=%3Atitle%3A%20covid-19%20or%20%3Atitle%3A%20covid19%20or%20%3Atitle%3A%202019-ncov%20or%20%3Atitle%3A%20sars-cov2%20or%20%3Atitle%3A%20severe%20acute%20respiratory%20syndrome%20or%20%3Atitle%3A%20middle%20eastern%20respiratory%20syndrome%20and%20%3Atitle%3A%20Arbidol%20OR%20%3Atitle%3A%20atazanavir%20OR%20%3Atitle%3A%20Azithromycin%20OR%20%3Atitle%3A%20Baloxavir%20OR%20%3Atitle%3A%20Baricitinib%20OR%20%3Atitle%3A%20Bevacizumab%20OR%20%3Atitle%3A%20Chloroquine%20OR%20%3Atitle%3A%20Colchicine%20OR%20%3Atitle%3A%20Darunavir%20OR%20%3Atitle%3A%20Emtricitabine%20OR%20%3Atitle%3A%20Enisamium%20OR%20%3Atitle%3A%20Favipiravir%20OR%20%3Atitle%3A%20Fingolimod%20OR%20%3Atitle%3A%20Ganciclovir%20OR%20%3Atitle%3A%20Hydroxychloroquine%20OR%20%3Atitle%3A%20Indinavir%20OR%20%3Atitle%3A%20Lopinavir%20%3Atitle%3A%20Mycophenolic%20OR%20%3Atitle%3A%20Nelfinavir%20OR%20%3Atitle%3A%20Niclosamide%20OR%20%3Atitle%3A%20Nitazoxanide%20OR%20%3Atitle%3A%20Novaferon%20OR%20%3Atitle%3A%20Oseltamivir%20OR%20%3Atitle%3A%20Pirfenidone%20OR%20%3Atitle%3A%20Quercetin%20OR%20%3Atitle%3A%20Remdesivir%20OR%20%3Atitle%3A%20Ribavirin%20OR%20%3Atitle%3A%20Ruxolitinib%20OR%20%3Atitle%3A%20Sirolimus%20OR%20%3Atitle%3A%20Sofosbuvir%20OR%20%3Atitle%3A%20Tocilizumab%20OR%20%3Atitle%3A%20Thymosin%20OR%20%3Atitle%3A%20Triazavirin%20OR%20%3Atitle%3A%20steroids%20OR%20%3Atitle%3A%20steroid%20or%20%3Atitle%3A%20glucocorticosteroid%20OR%20%3Atitle%3A%20glucocorticosteroids%20OR%20%3Atitle%3A%20corticosteroid%20OR%20%3Atitle%3A%20corticosteroids%20OR%20%3Atitle%3A%20cortisone%20OR%20%3Atitle%3A%20hydrocortisone%20OR%20%3Atitle%3A%20prednisone%20OR%20%3Atitle%3A%20prednisolone%20OR%20%3Atitle%3A%20methylprednisolone%20OR%20%3Atitle%3A%20dexamethasone%20OR%20%3Atitle%3A%20fludrocortisone%20OR%20%3Atitle%3A%20interferon%20OR%20%3Atitle%3A%20atorvastatin%20OR%20%3Atitle%3A%20rosuvastatin%20or%20%3Atitle%3A%20simvastatin%20OR%20%3Atitle%3A%20pravastatin%20OR%20%3Atitle%3A%20Fluvastatin%20OR%20%3Atitle%3A%20fluindostatin%20OR%20%3Atitle%3A%20lovastatin%20OR%20%3Atitle%3A%20pitavstat)

## PrePrints.org

Abstract contains:

- Covid-19 OR
- Covid19 OR
- Covid2019 OR
- Covid-2019 OR
- Covid OR
- 2019-ncov OR
- sars-cov-2 OR
- SARS OR
- MERS

AND Abstract contains:

- Arbidol OR atazanavir OR Azithromycin OR Baloxavir OR Baricitinib OR Bevacizumab OR Chloroquine OR Colchicine OR Darunavir OR Emtricitabine OR Enisamium OR Favipiravir OR Fingolimod OR Ganciclovir OR Hydroxychloroquine OR Indinavir OR Lopinavir
- Mycophenolic OR Nelfinavir OR Niclosamide OR Nitazoxanide OR Novaferon OR Oseltamivir OR Pirfenidone OR Quercetin OR Remdesivir OR Ribavirin OR Ruxolitinib OR Sirolimus OR Sofosbuvir OR Tocilizumab OR Thymosin OR Triazavirin OR steroids OR steroid
- glucocorticosteroid OR glucocorticosteroids OR corticosteroid OR corticosteroids OR cortisone OR hydrocortisone OR prednisone OR prednisolone OR methylprednisolone OR dexamethasone OR fludrocortisone OR interferon OR atorvastatin OR rosuvastatin
- simvastatin OR pravastatin OR Fluvastatin OR fluindostatin OR lovastatin OR pitavstatin OR convalescent OR reconvalescent

## ChinaXiv

Search terms are:

- COVID-19 virus: 新型冠狀病毒 (full term) or 新冠病毒 (short term)
- COVID-19 pneumonia: 新型冠狀病毒肺炎 (full term) or 新冠肺炎 (short term)
- SARS: 嚴重急性呼吸綜合症
- MERS: 中東呼吸綜合症
